# Black-Box Gastrointestinal Tract—Needs and Prospects of Gaining Insights of Fate of Fat, Protein, and Starch in Case of Exocrine Pancreatic Insufficiency by Using Fistulated Pigs

**DOI:** 10.3390/nu9020150

**Published:** 2017-02-16

**Authors:** Anne Mößeler, Josef Kamphues

**Affiliations:** Institute for Animal Nutrition, University of Veterinary Medicine Hannover, Foundation, Bischofsholer Damm 15, D-30173 Hanover, Germany; josef.kamphues@tiho-hannover.de

**Keywords:** animal model, exocrine pancreatic insufficiency, fistulated animals, prececal digestibility, protein, starch

## Abstract

Exocrine pancreatic insufficiency (EPI) results in the maldigestion and malabsorption of nutrients. The digestive processes in humans and other monogastric species like rat and pig are characterized by a predominantly enzymatic digestion within the small intestine and microbial fermentation located in the hindgut. For protein, it is doctrine that only prececally absorbed amino acids can be transferred to the amino acid pool of the host, while postileal absorption of nitrogen-containing compounds occurs mainly in the form of ammonia, being a burden rather than a benefit for the organism. The pig is an established animal model for humans to study digestive processes. As digestion is markedly impaired in case of EPI the use of an appropriate animal model to study the effects of this disease and to optimize treatment and dietetic measures is of special interest. By using an animal model of experimentally-induced EPI allowing differentiating between digestive processes in the small as well as in the large intestine by use of ileo-cecal fistulated animals, marked effects of EPI on prececal digestion of starch and protein could be shown. The data indicate that estimation of digestibility of nutrients over the entire digestive tract results in a distinct overestimation of enzymatic digestion of starch and protein. Therefore, this model clearly shows that protein and starch digestion are significantly reduced in case of EPI although this cannot be detected on a fecal level. As postileal fermentation of starch is associated not only with energy losses but also with intensive gas production, this is of special interest to minimize meteorism and improve wellbeing of patients.

## 1. Background—Digestive Processes in the Gastrointestinal Tract

The gastrointestinal tract (GIT) is highly complex, consisting of several organs which work in a precisely adjusted manner in a healthy individuum. Besides the salivary glands, teeth and stomach, the small and large intestine, and liver, the pancreas plays a very important role in the process of digestion [1,2]. The apparent digestibility of feed and food can be measured by quantification of intake and fecal excretion. In human nutrition, digestibility is of special interest in the case of diseases of the digestive tract but often the clinical symptoms (osmotic diarrhea, voluminous stool, etc.) are more relevant than the precise values of digestibility. There is profound knowledge from animal nutrition in monogastric species (to which humans belong as well) regarding the extent of digestion in the prececal (small intestine) or postileal (hindgut) parts of the GIT [1,2,3,4,5], but knowledge in humans is scarce. 

**Figure 1:**
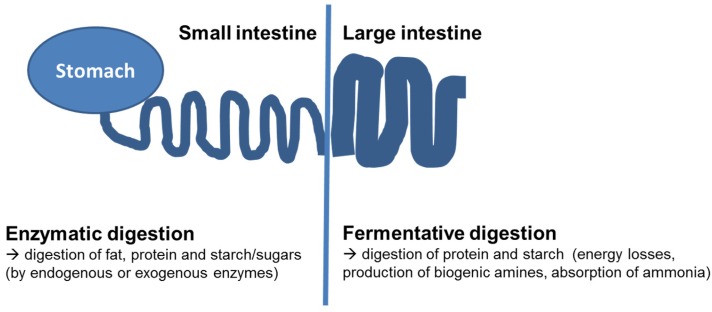
Schematic gastrointestinal tract of humans and monogastric animals and localization of enzymatic or fermentative digestion.

The differentiation as to whether nutrients are digested and absorbed in the small intestine or in the hindgut is of great relevance as the digestion processes in the small intestine are based mainly on enzymatic digestion—with a species-dependent proportion of microbial digestion, whereas the digestive processes in the hindgut are based on bacterial degradation (see Figure 1). Although this microbial fermentation allows the host to generate energy (e.g., from fermentative degradation of resistant starch or fiber but also of prececally undigested proteins and peptides), there is consensus that only prececally digested proteins can be included in the amino acid pool of the host and thereby contribute to amino acid supply as there is no relevant absorption of amino acids from the hindgut [6,7,8]. The fate of protein in the hindgut is caused by absorption of ammonia, which is produced by bacterial degradation [8]. Ammonia can be used for producing nonessential amino acids by the host but is also metabolized to urea and excreted via urine. However, the ammonia or other N-containing compounds are used for microbial protein synthesis [9]. Nonetheless, in monogastric species, these microbial proteins cannot be absorbed and used in intermediate metabolisms but are excreted via feces [8]. For some amino acids (especially methionine), the bacterial synthesis results in a netto synthesis in the hindgut. Therefore, using fecal samples for analysis results in an underestimation of digestibility and nutritive value of food and feed [10]. Thus, determining prececal digestibility of amino acids is standard in animal nutrition worldwide, to evaluate the nutritive value of protein feedstuffs and requirements (of poultry, horses, and pigs) and recommendations on amino acid supply are given in prececal digestible amino acids [8,11,12]. 

One established method used worldwide to differentiate the localization of digestive processes is the use of fistulated animals [13,14]. As the pig shows good similarity of the digestive function with humans, it is assumed to be an excellent model for studying digestive processes [15,16,17,18,19,20,21]. The need for suitable animal models is especially high in case of impaired digestion due to diseases of the GIT. A disease that has been intensively studied in the animal model of the pig is exocrine pancreatic insufficiency (EPI). A complete EPI can be induced by ligation of the pancreatic duct. The pancreatic duct ligated (PL) pig is an animal model that was established decades ago and is still used today [22,23,24,25,26,27,28,29,30,31,32,33,34,35,36,37,38]. The lack of pancreatic secretion results in a distinct reduction in digestibility of diverse nutrients, mimicking the situation in human patients. As in human patients [39,40], the increased excretion of fat via feces (steatorrhea) is the most typical symptom of EPI in pigs [29]. Comparable to humans, there is no relevant fecal excretion of starch in EPI-pigs, even no pancreatic enzymes are substituted and diet is rich in starch [33,34,41]. These findings led to the assumption that starch digestion is not relevantly affected in EPI-patients and consequently dietary recommendations do not include any recommendations specifically regarding amylase substitution or limitation of starch. Nonetheless, some authors state that pancreatic enzyme replacement therapy (PERT) is recommended even when diet is low in fat but rich in starch [42,43]. Protein digestion is only moderately impaired in EPI-patients as well, taking into account the fecal digestibility. Therefore, the dietary recommendations for EPI-patients do not include protein intake or protease uptake in general. Nonetheless, a recently published guideline for nutrition states that there is a higher protein need in EPI-patients and adjustment of protein intake is recommended [44].

To characterize the digestive processes at different localizations of the GIT precisely, fistulated animals are of great value. The digestion within the small intestine is characterized by activity of pancreatic enzymes and brush border membrane enzymes, resulting in an efficient digestion of fat, protein, and starch in healthy individuals. Bile acids contribute to emulsification of fat to optimize the absorption. The digesta entering the hindgut consists of all nutrients that escaped digestion in the small intestine as well as compounds of endogenous origin (e.g., mucus, scaled off cells). Due to the activity of the microflora in the hindgut, most nutrients entering the hindgut are fermented efficiently [1,2,45]. Besides fiber, the intestinal microflora degradates resistant starch (starch that was not digested in the small intestine) as well as protein. The microbial fermentation results in the production of short chain fatty acids which are absorbed and are of energetic value for the host [46]. In contrast, the intestinal gas production (CO_2_, H_2_, CH_4_) might negatively affect the wellbeing of the host by meteorism, bloating, and flatulence [47]. Nitrogen (N) compounds are absorbed from the hindgut mainly in the form of ammonia. Therefore, an absorption of N from the hindgut is more of a burden for the host than a benefit [48]. The aim to maximize prececal digestibility of dietary protein is additionally based on the finding that several products of protein fermentation (like ammonia, phenolic and indolic compounds, as well as hydrogen sulfide) have a toxic potential [49]. Therefore, protein fermentation in the hindgut results in toxic luminal compounds that affect epithelial cell metabolism and barrier function [49]. There are manifold interactions between protein and carbohydrate fermentation in the hindgut, modifying the produced metabolites of protein fermentation relevantly. Presence of fermentable carbohydrates results in a higher bacterial protein synthesis, leading to lower concentrations of protein-derived fermentation end products [50].

Put simply, the digestion within the small intestine can be classified as being mainly enzymatic (digestion by activity of digestive enzymes from the host) with a small proportion of microbial degradation, while the digestion in the hindgut is exclusively due to fermentation (see Figure 1). 

## 2. Use of Ileo-Cecal Fistulated Pigs in Research on Diverse Effects of Exocrine Pancreatic Insufficiency (EPI) on Digestive Processes and Optimization of Treatment and Dietetic Measures

**Figure 2:**
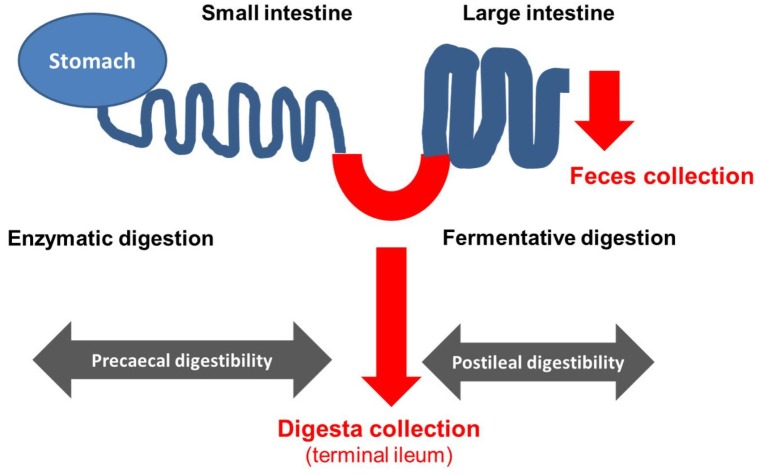
Scheme of gastrointestinal tract of an ileo-cecal fistulated animal and the matrix available for analysis to determine prececal (by analysis of ileal digesta) as well as postileal digestibility and total tract digestibility (by analysis of feces).

The use of animals fitted with an ileo-cecal fistula (see Figure 2) allows differentiation between digestion in prececal parts of the GIT which are mainly but not exclusively based on action of enzymatic activity of the animal and postileal digestion (only fermentative digestive processes). The technique of an ileo-cecal re-entrant fistula in pigs was first described by in 1973 by Easter and Tanksley [51], and Hazem and Drochner [52]. 

The use of ileo-cecally fistulated animals allows characterization and quantification of the digestive processes in the small and large intestine in detail. By this, it is possible to differentiate between the capacity of the enzymatic digestion (small intestine) as well as the fermentative digestion (hindgut). This would not be possible by using animals with ileo-rectal anastomosis which is another established method to determine ileal digestibility [53,54]. 

A great advantage of using fistulated animals to investigate digestive processes in the case of impaired digestion due to EPI is the possibility of testing the efficacy of different enzyme preparations used for pancreatic enzyme replacement therapy under totally standardized conditions [31,35,55,56,57]. Furthermore, different diets and dietetic measures can be comparatively tested as well [58,59,60,61]. By using fistulated animals in a randomized cross-over design, it is possible to minimize individual effects of the animal. 

Several studies have been performed using the animal model of the adult pancreatic duct ligated, ileo-cecal fistulated (mini)pig (see Table 1) [29,34,41,57,58,59,60,61,62,63,64,65,66,67,68,69]. This model enables the studying of the effects of different enzyme products, different treatments (enzyme dosages, different meals and different combinations of enzymes and diets) under standardized conditions, and total control of nutrient uptake.

The results generated in the animal model of the pancreatic duct ligated and ileo-cecal fistulated animals (see Table 2) allow a differentiation between residual extrapancreatic digestive capacity within the small intestine and the fermentative capacity of the large intestine in case of EPI [34].

By comparing the data of healthy controls and PL-pigs without pancreatic enzyme supplementation the impact of exocrine pancreas on digestion can be quantified. The largest effect of EPI was found for apparent crude fat digestibility which reached maximum values of 31.5% over the entire GIT in PL-pigs, whereas in healthy controls values up to 97.4% were observed. For apparent total tract protein digestibility, the values of controls varied around 90%, while in PL-pigs without enzyme supplementation the values did not exceed 67.5%, with most values around 50%. Only for starch digestibility no clinical relevant differences were observed by analysis of the feces when controls and PL-pigs were compared, both groups of animals reaching values of around 99% (see Table 2). 

Regarding fat, there was no large difference between prececal digestibility determined by use of ileo-cecal fistulated animals and coefficient of absorption over the entire GIT. Interestingly, in some studies the value for total tract digestibility was lower than at the ileal level [41,62], indicating a postileal fat synthesis due to bacterial activity and an effect of scaled off cells. The missing compensatory digestive capacity of the hindgut for fat digestion results in the massive fecal fat excretion which is a typical symptom of EPI [39]. 

For protein, there was only a relatively small difference between prececal and total tract apparent digestibility in control pigs, with values ranging between 79.1–82.3% at the ileal level and 87.5%–92.2% at the fecal level. For PL-pigs, the mean values were markedly lower (30.8% at ileal level and 52.1% at fecal level). Interestingly, the relative effect of postileal absorption of nitrogen in the hindgut was much greater in PL-pigs compared to controls, indicating an increased burden of the liver (formation of urea) and kidney (excretion) as discussed by Tabeling [41]. 

While for fat and protein, the analysis of feces would have resulted in findings indicating reduced digestibility of the nutrients in PL-pigs (comparable to findings in human patients suffering from EPI) [70,71], this would not have been the case for starch. While starch digestion was almost complete even in PL-pigs receiving no pancreatic enzymes, which is in agreement with human patients suffering from EPI in which also no fecal starch excretion is found in most cases [72], the prececal digestibility reached only values around 70% with values differing between 50% and 93%. The prececal digestibility of starch is dependent on botanical origin but also on thermal treatment of the starch [59,60,73]. From these values, it can be calculated that quite high amounts of starch are digested in the hindgut in EPI-patients not treated with amylolytic enzymes. As indicated by in vitro studies [73] the fermentation of starch by bacteria from digesta collected at the terminal ileum is accompanied by an intensive gas production (up to 160 mL of gas/g fermented starch). These findings underline the need to use fistulated animals to determine prececal digestibility of starch, to take into account the localization of the digestion of nutrients and to differentiate between enzymatic and fermentative digestion. Furthermore, these results clearly show that treatment of EPI-patients should not only be focused on lipase supplementation. The use of multienzyme products containing amylolytic enzymes is recommended to optimize prececal digestibility of starch [43].

To point out the clinical relevance of the reduced digestibility for energy supply of the patient, the data generated by Stefaniak [61] in the animal model of the pancreatic duct ligated ileo-cecal fistulated pigs were used to calculate the digestible energy of the nutrients. The digestible energy is a standard parameter in animal nutrition and is calculated by using the gross caloric value of the ingested nutrients and the apparent (total tract) digestibility [1,2]. As the postileal fermentation of starch contributes to the energy supply of the host but is also accompanied by negative side effects and is therefore unintended, the data of prececal digestibility were used for this exemplary calculation (condoning energetic value of fermented starch and protein is not taken into account). As the fate of protein from the hindgut is supposed to result mainly from ammonia absorption [8,41], the use of the prececal digested protein for calculation purposes seems reasonable although formation of short chain fatty acids may contribute to energy supply. If only digestibility of fat is of interest no collection of ileal digesta is necessary. However, if ileal digesta is collected to determine prececal digestibility of protein and starch, the use of ileal digesta for determining fat digestibility is also possible. For fat, the use of prececal digestibility can be judged as well as there is no relevant fat absorption in the hindgut in most studies or even a low microbial fat synthesis, resulting in an underestimation of fat digestion. 

When taking into account the prececal digestibility rate of the nutrients, the digestible energy can be calculated for each gram of ingested nutrient. While it is possible to estimate the digestible energy per gram ingested nutrient in healthy individuals quite well (as prececal digestion is almost complete) this is much more difficult in EPI-patients. As the digestibility is markedly reduced, the digestible energy that can be generated is much lower than calculated from the energy intake. It can be speculated that the higher energy requirements that are postulated for some diseases associated with EPI (e.g., cystic fibrosis [74]) may be, at least to some extent, based on the reduced efficiency of digestion or lower energy utilization of the ingested nutrients. In Table 3, the data of prececal digestibility of nutrients generated in ileo-cecal fistulated pigs are given for healthy controls as well as for pigs with experimentally-induced EPI. The latter group was fed the diet twice, one time without any treatment with enzymes and on another occasion with pancreatic enzyme treatment therapy (PERT) using a high dosage of a porcine pancreatin product. While the prececal digestibility of starch and fat reached very high levels in controls (98.8% and 93.9%, respectively) the apparent prececal digestibility of protein was somewhat lower (~80%). This is due to the endogenous losses (e.g., N-containing secretions like mucus or bile and scaled off cells); therefore, the real digestibility of protein can be assumed to be higher [14]. The pigs with experimentally-induced EPI showed markedly reduced prececal digestibility rates, only 22.7% of the fat and 31.9% of the ingested protein were absorbed prececally. For starch, the observed prececal digestibility rate was higher (65.7%) but was significantly lower than in controls. The relatively high prececal digestibility rate of starch in pigs lacking complete exocrine function is assumed to be due to amylolytic enzymes of saliva as well as microbial fermentation. When PL-pigs received PERT, the digestibility rates markedly improved with values reaching 90% to 96% of those of the controls. It is worth mentioning that, under these conditions, the fat digestibility was 3.7 times higher than in pigs without PERT (see Table 3). On the basis of the prececal digestibility, the digestive energy (DE) per g of nutrient intake was calculated. While for controls the sequence was fat > starch > protein as expected (reflecting the high gross energy of the fat), the PL-pigs without PERT could generate most energy per gram nutrient from starch; therefore, the sequence was starch > fat > protein. In PL-pigs receiving PERT, the sequence of digestible energy density was, like in the controls, fat > starch > protein. The data indicate that PEI patients not treated with PERT can only generate a rather low proportion of energy from the diet compared to the controls. For fat these individuals reach only 24% of the digestible energy value a healthy individual can generate and even for starch the energy uptake is about only 67% of the controls (see Table 3). 

Based on these data, the amount of nutrient intake required to generate 1000 kJ of digestible energy can be calculated. To simplify the calculation it was assumed that the energy is generated only from one single nutrient. While a healthy control pigs needs to ingest only 27.6 g of fat, 58.8 g starch or 73 g of protein to generate 1000 kJ digestible energy, a PL-pig without PERT needs to ingest much higher amounts (88.5 g of starch, 114 g of fat or 182 g of protein). By using an efficient PERT, it is possible to generate the energy by an intake of meals only slightly higher (max + 12%) than in the controls. 

When calculated on the level of DE, the food intake requirements of these patients is distinctly higher; taking into account the reduced energetic value of the diet in these patients, this might be another relevant factor.

Although this calculation has some limitations as the digestibility of nutrients may vary depending on source and treatment of the food [59,60,73] and postileal fermentation of starch and protein was not included, the results impressively show the marked effects of EPI on the capability to generate digestible energy from food. The data indicate that the nutrient intake needs to be much higher in EPI-patients to achieve a balanced energy status compared to the controls if there is no proper PERT. Furthermore, the data clearly show that starch as well as protein digestion are impaired considerably. Therefore, the need for using enzyme products containing amylolytic as well as proteolytic enzymes is mandatory in EPI-patients. The impressive increase of nutrient digestibility due to PERT was seen in several experimental studies using the PL-pig as an animal model for human EPI [23,31,33,37,41,50,53,54,61,62,63,64,65,66,67,68,69]. In juvenile individuals with EPI a proper PERT is indispensable to prevent growth retardation [30,36,37].

To summarize, the results generated in the animal model of the ileo-cecal fistulated pig clearly show that the enzymatic (prececal) digestion of protein and starch is markedly overestimated when feces are used as samples. As feces are the only matrix available in human patients, the limitations of this matrix should be addressed [76]. As there is good evidence that the data generated in animal studies can be transferred to human situation, these data are highly valuable to optimize therapy and nutritional measures in EPI-patients. Taking into account the high compensative digestive capacity of the hindgut for starch and protein [34,45], it becomes obvious that testing amylolytic and proteolytic enzymes can be done accurately only by determining prececal digestibility of starch and protein. Experimental studies in this animal model have repeatedly shown that there is no correlation between the effects of different sources of starch in healthy control animals and animals with experimentally-induced EPI [59,60]. In a recent study, it was shown that high caloric drinks which are highly digestible in healthy controls were markedly less digestible in PL-pigs [58]. From these findings, it can be concluded that dietetics and dietetic measures need to be tested and proven in a suitable animal model. Therefore, the combination of the experimentally-induced EPI and the ability of measure the prececal digestibility in this model offers a great chance to optimize PERT as well as diets and drinks designed for patients with maldigestion [55,58].

Although the limitations of every method should be evaluated critically in research, it needs to be emphasized that the animal model of the pancreatic duct ligated pig has been established justifiedly as the gold standard to study EPI and to test the efficacy of substituted enzymes [29,50,52,53,54,55,56,57]. This model enables the study of the manifold effects of EPI on body composition and quantification and sampling of diverse tissues (when slaughtering the animals after a defined period of EPI) [36,38]. Nevertheless, for studying digestive processes and comparing different dietetic measures the possibility to test several diets, enzymes, or dosages within one animal is of greatest value. The greatest advantage of using adult fistulated animals is the ability to perform comparative repeated trials in each animal allowing differentiation between digestive processes in the small intestine (mainly, although not completely a result of the activity of digestive enzymes from the host) and in the large intestine (exclusively microbial fermentation). This enables evaluation of the energetic and nutritive value of the ingested food more precisely. For example, the same amount of starch digested (a) completely in the small intestine and (b) 50% in the small intestine and 50% in the large intestine does not only result in a lower energy supply for the host (a reduction of 15% of energy supply is assumed in pigs and it can be assumed that this level can be taken for granted in humans as well) but also in undesired effects like intestinal gas production [77,78]. The gas production can reach levels up to 160 mL/g starch as observed in in vitro studies using ileal digesta of pigs with experimentally-induced EPI [69]. As there is no fecal starch excretion, even in PL-pigs not treated with enzymes, the exclusive analysis of feces would have resulted in an overestimation of the nutritive value (energy supply). Associated negative side effects (meteorism, flatulence, and fermentative diarrhea) with starch maldigestion can be reduced if PERT is done by using pancreatic enzyme products containing amylolytic enzymes.

Even the fistulation is a surgical intervention and taking care of these animals is much more challenging and labor-intensive compared with non-fistulated ones, it needs to be emphasized that the animals can be kept in good general condition for a long time (up to eight years). 

The use of adult fistulated animals offers the advantage to test different foods and diets as well as enzyme products in one animal comparatively (minimizing individual effects) and to reduce the number of animals used in these trials [79]. One disadvantage might be that the location of GIT from which samples can be taken is fixed. Slaughtering trials allow to take samples from all parts of the digestive tract and furthermore to sample tissue from diverse regions of the whole animal body (liver, bones, fat tissue, etc.) in order to quantify the effect of feeding measures or treatment on the whole organism [33,36]. However, the individual variation is higher as no investigations can be undertaken to test the effects of different diets or enzymes comparatively, and due to the nature of these final studies, the number of animals needed is higher. 

## 3. Conclusions

To conclude, the use of fistulated animals offers the ability to precisely determine the prececal digestibility of diverse nutrients, thus enabling deeper insights into digestive processes. Without the knowledge of these animal studies, the extent of impairment of protein and starch digestion in case of EPI could not be detected and studied in detail. Therefore, this technique contributes greatly to a better understanding of the manifold effects of EPI on digestion processes and the resulting therapeutic and dietetic measures [80]. From the results of these animal studies, it can be concluded that for therapy of patients suffering from EPI the use of enzymes containing lipolytic, proteolytic as well as amylolytic enzymes is crucial to optimize digestive processes and to minimize negative side effects. 

## Figures and Tables

**Table 1:** Composition of the diets (% of dry matter) used in different studies with adult pancreatic duct ligated, ileo-cecal fistulated (mini)pigs.

Starch	Crude Fat	Crude Protein	Author
60.0	9.01	12.8	Tabeling (Starch Diet) [41]
56.2	2.21	21.2	Mandischer [62]
41.2	29.7	15.4	Stefaniak [61]
40.0	19.0	18.3	Kammlott [57]
39.0	32.7	14.9	Koch [63]
37.8	28.3	15.6	Kalla [64]
35.8	31.3	14.6	Classen [65]
33.4	34.9	14.9	Karthoff [66]
27.3	34.2	16.3	Heldt [67]
27.1	33.5	16.7	Fuente-Dege [68]
26.6	32.2	15.9	Tabeling (Fat Diet) [41]
24.1	30.1	14.4	Fassmann [69]

**Table 2:** Apparent prececal (prc.) and total tract (total) digestibility rates (%) in pancreatic duct ligated (PL)-pigs without pancreatic enzyme replacement therapy and control-pigs.

Starch		Crude Fat		Crude Protein		Author	
Prc.	Total	Prc.	Total	Prc.	Total		
61.9	99.3	9.30	-6.60	29.6	56.7	Tabeling (Starch Diet) [41]	PL-pigs
63.5	N.A.	28.6	8.43	30.5	67.5	Mandischer [62]	
65.7	N.A.	22.7	28.1	31.9	48.4	Stefaniak [61]	
72.6	N.A.	19.4	23.3	30.9	60.0	Kammlott [57]	
61.1	N.A.	16.2	26.2	28.2	47.9	Koch [63]	
50.0	N.A.	14.0	22.0	24.8	44.9	Kalla [64]	
61.4	98.4	25.1	23.1	33.7	35.1	Classen [65]	
75.4	N.A.	29.7	29.2	32.3	48.6	Karthoff [66]	
63.8	N.A.	29.0	17.8	33.7	54.1	Heldt [67]	
75.6	N.A.	29.9	28.2	40.5	54.1	Fuente-Dege [68]	
87.7	99.1	43.0	31.5	27.3	56.9	Tabeling (Fat Diet) [41]	
92.5	N.A.	32.5	14.5	26.1	49.9	Fassmann [69]	
**94.6–99.7**	**~100**	**88.2–98.3**	**81.3–97.4**	**79.1–82.3**	**87.5–92.2**	**Control-pigs (all studies)**	

N.A.: not assessed.

**Table 3:** Prececal digestibility rates of nutrients in healthy control pigs and PL-pigs (without or with pancreatic enzyme replacement therapy (PERT)) and the derived digestible energy (DE) per gram of nutrient intake and amount of nutrient intake needed to generate 1000 kJ DE; the values in brackets represent the relative values (the controls being set to 100).

		Control	PL-pig	
			Without PERT	With PERT ^#^
Prececal digestibility (%) *	Fat	93.9	22.7 (24.2)	84.0 (89.5)
	Protein	79.9	31.9 (39.9)	73.9 (92.5)
	Starch	98.8	65.7 (66.5)	94.8 (96.0)
Digestible energy (DE; kJ) per g ingested nutrient **	Fat	36.2	8.74 (24.1)	32.3 (89.2)
	Protein	13.7	5.49 (40.1)	12.7 (92.7)
	Starch	17.0	11.3 (66.5)	16.3 (95.9)
Amount of nutrient intake needed (g) to generate 1000 kJ DE ***	Fat	27.6	114 (413)	31.0 (112)
	Protein	73.0	182 (249)	78.7 (108)
	Starch	58.8	88.5 (150)	61.3 (104)

^#^ 300,000 International Units (according to FIP; Federation-Internationale-Pharmaceutique) IU lipase; 17,332 IU protease; 306,455 IU amylase/meal containing 66.5 g crude fat, 34.4 g crude protein, 92.3 g starch equivalent to 4511 IU lipase/g fat; 504 IU protease/g crude protein; 3320 IU amylase/g starch. * for fat and protein, the values represent the apparent digestibility (due to endogenous losses). ** gross energy values used for calculation (kJ/g): fat: 38.5; protein: 17.2; starch 17.2 (according to Lückerath & Müller 2011 [75]). *** Assumption: 1000 kJ are generated exclusively by uptake of one isolated nutrient.
